# A novel neutrophil extracellular trap signature to predict prognosis and immunotherapy response in head and neck squamous cell carcinoma

**DOI:** 10.3389/fimmu.2022.1019967

**Published:** 2022-09-23

**Authors:** Qilin Li, Weimin Chen, Qiuhui Li, Jing Mao, Xin Chen

**Affiliations:** ^1^ Department of Stomatology, Tongji Hospital of Tongji Medical College, Huazhong University of Science and Technology, Wuhan, China; ^2^ School of Stomatology, Tongji Medical College, Huazhong University of Science and Technology, Wuhan, China; ^3^ Hubei Province Key Laboratory of Oral and Maxillofacial Development and Regeneration, Wuhan, China; ^4^ State Key Laboratory Breeding Base of Basic Science of Stomatology (Hubei-MOST) and Key Laboratory for Oral Biomedicine of Ministry of Education, School and Hospital of Stomatology, Wuhan University, Wuhan, China; ^5^ Department of Oncology, Tongji Hospital, Tongji Medical School, Huazhong University of Science and Technology, Wuhan, China

**Keywords:** head and neck squamous cell carcinoma, neutrophil extracellular traps, immune cells (ICs), immunotherapy, prognosis

## Abstract

Head and neck squamous cell carcinoma (HNSCC) is one of the most common malignant cancers, and patients with HNSCC possess early metastases and poor prognosis. Systematic therapies (including chemotherapy, targeted therapy, and immunotherapy) are generally applied in the advanced/late stages of HNSCC, but primary and acquired resistance eventually occurs. At present, reliable biomarkers to predict the prognosis of HNSCC have not been completely identified. Recent studies have shown that neutrophil extracellular traps (NETs) are implicated in cancer progression, metastasis and cancer immune response, and NET-related gene signatures are associated with the prognosis of patients with several human cancers. To explore whether NET-related genes play crucial roles in HNSCC, we have performed systematic analysis and reported several findings in the current study. Firstly, we identified seven novel NET-related genes and developed a NET-score signature, which was highly associated with the clinicopathological and immune traits of the HNSCC patients. Then, we, for the first time, found that NIFK was significantly upregulated in HNSCC patient samples, and its levels were significantly linked to tumor malignancy and immune status. Moreover, functional experiments confirmed that NIFK was required for HNSCC cell proliferation and metastasis. Altogether, this study has identified a novel NET-score signature based on seven novel NET-related genes to predict the prognosis of HNSCC and NIFK has also explored a new method for personalized chemo-/immuno-therapy of HNSCC.

## Introduction

Head and neck cancer (HNC) ranks sixth in terms of malignancy worldwide, and about 90% of HNCs are classified as head and neck squamous cell carcinomas (HNSCC) (1). HNSCC possesses a high incidence of cervical lymph node metastases, increased capacities of invasive and recurrence, and contributes to the poor prognosis (2, 3). The main treatment options for HNSCC include surgery, chemotherapy, radiotherapy, molecular targeted therapy, and multimethod in conjunction with surgical excision of tumor tissue, but the effectiveness of these approaches is limited due to tumor heterogeneity (4, 5).

Cancer immunotherapy is based on harnessing the immune system to detect and eliminate tumor cells, and the field of cancer immunotherapy has been growing with an increasing rate in modern oncology since it was first mentioned in 1985 (6–8). Active immunotherapy, passive immunotherapy, and immune checkpoint blockade are the major strategies of cancer immunotherapy (9, 10). For HNSCC immunotherapy, two immune checkpoint blockade agents, pembrolizumab and nivolumab, have been applied in clinical trials for patients with platinum-refractory HNSCC (11, 12). However, most HNSCC patients are non-responders and have acquired drug resistance (13–15). Recent studies have indicated that cancer immunotherapy may be hindered by immunosuppressive cells of the tumor microenvironment (TME), leading to the failure of antitumor immunity (16, 17).

Neutrophils are the most abundant immune cells in the TME, and increased neutrophil infiltration and high neutrophil-to-lymphocyte ratios were reported to be associated with poor patient outcomes of the patients with HNSCC (18–20). In activating neutrophils, DNA fibers decorated with histones and antimicrobial proteins found originally within neutrophil granules are released as neutrophil extracellular traps (NETs) (21, 22). NETs have been found as a new form of innate immunity and mediate the response of the host as a first line of defense (23). The development of NETs is a potential mechanism that contributes to tumor progression. Additionally, tumor cells can also escape immune surveillance through NETs (24). While the pro-oncogenic evidence of NETs is growing, the role of NETs in cancer immunotherapy remains unclear, particularly in HNSCC.

In this study, we have developed a novel NETs-score signature consisting of seven NETs-related genes, and we have found that NETs-score could reflect the response of HNSCC patients to chemotherapy and immunotherapy. Lastly, we have further identified the NET-related gene NIFK as a potentially carcinogenic factor for patients with HNSCC.

## Methods and material

### HNSCC database handling

HNSCC-related clinical information has been downloaded and collected from the TCGA database (519 patients, https://xenabrowser.net/) and GEO database (ID: GSE41613 n = 97, GSE42743 n = 103, GSE65858 n = 270, https://www.ncbi.nlm.nih.gov/geo/). Transcriptional profiles of 989 HNSCC patients were obtained from four cohorts, and the patients with insufficient OS information were excluded (**Table S1**).

The Affymetrix and Illumina platforms were used to generate raw data from the TCGA and GEO databases. Background correction and normalization are achieved using a robust multi-chip averaging (RMA) algorithm. The TCGA database provides RNA sequencing data. The fragment per kilobase (FPKM) values were converted to transcripts per kilobase (TPM) values with signal intensities similar to the RMA treatment.

### Establishment of NET enrichment score

According to a recent study, we obtained a list of published NET gene sets and the descriptions of the gene sets (25–27) (**Tables S2, S3**). This NET-related gene set has a total of 69-gene with NET-initial biomarkers. We first performed univariate cox analysis to screen out the NET gene set associated with the prognosis of HNSCC patients for subsequent enrichment score calculation. NET-enrichment-score was calculated with single sample Gene Set Enrichment Analysis (ssGSEA) for HNSCC patients using the NET gene set associated with prognosis for further analysis.

### Establishment of a NET-related signature

We employed Spearman correlation analysis to identify genes that were significantly positively correlated with NET-enrichment-score (correlation coefficient >0.4 and P-value <0.05, termed NET-related genes) and were selected for further analysis. Subsequently, we performed univariate Cox regression analysis to identify NET-related genes associated with the prognosis of HNSCC patients (*P*-value <0.05). We then screened out more valuable NET-related genes with prognostic potential by applying machine learning algorithms through the R “CoxBoost” and “randomForestSRC” packages. The NET-related signature named NET-score was constructed from the list of NET-related genes with prognostic potential and weighted by their estimated regression coefficients in the Lasso regression analysis. Finally, we verified the prognostic evaluation performance of the NET-score. We estimated the NETs-score of 519 patients in the TCGA–HNSCC dataset, and then divided the patients into high and low NET-score groups based on the *P* value of the best cut-off. Kaplan–Meier curve analysis of the association between OS and NET-score. Time-ROC was used to validate the efficiency and accuracy of the NET-score for 1-year, 3-year, and 5-year prognosis prediction. Univariate and multivariate cox regression analyses were performed on the NET-score.

### Genomic alteration

Somatic mutation and somatic copy number variation (CNV) data were collected from the TCGA dataset. Genomic Identification of Important Targets in Cancer (GISTIC) analysis was used to assess genomic signatures. The CNV landscape and the copy number gain or loss of amplified or deleted peaks were assessed by GISTIC 2.0 analysis (https://gatk.broadinstitute.org).

### Assessing the immunological profile of the TME

We first used the ESTIMATE (The Estimation of Stromal and Immune cells in Malignant Tumor tissues using Expression) algorithm to estimate the abundance of immune cells and the infiltration level of stromal cells in HNSCC tumor tissue, which were reflected by immune score, stromal score, and estimated score, respectively. The Tumor Immune Estimation Resource2.0 (TIMER2.0, http://timer.cistrome.org/) web server was used to comprehensively analyze the level of immune-infiltrating cells in HNSC. Then, the relative proportions of 10 immune cells in the tumor were estimated using the MCPcounter algorithm. The infiltration levels of the 28 immune cells were represented by the enrichment scores based on the corresponding features. Enrichment scores were calculated using Single-Sample Genomic Enrichment Analysis (ssGSEA) implemented using the R Genomic Variation Analysis (GSVA) package. The response of HNSCs to anti-PD1 and anti-CTLA4 therapy was assessed by the submap algorithm. Response to anti-immune checkpoint therapy was assessed by the TIDE algorithm (28).

### Functional annotation of differently expressed NET-related genes

The Gene Ontology (GO) and Kyoto Encyclopedia of Genes and Genomes (KEGG) gene sets were downloaded from the MSigDB database (29). Gene Set Enrichment Analysis (GSEA) and Gene Set Enrichment Analysis (GSEA) and GSVA are implemented by the clusterProfiler R package and the GSVA R package (30).

### Prediction of drug response

We first used the Pharmacogenomics Data of Cancer Drug Sensitivity Genomics (GDSC, https://www.cancerrxgene.org/) to predict drug susceptibility in the included HNSCC cases (31). Drug responses were calculated with the oncoPredict R software package for drug sensitivity (32).

### Plate clone formation assay

The Cal27 and SCC25 cells were digested and then resuspended in serum-free medium, and the cells were seeded into a 6-well culture plate at a density of 10^3^ cells per well. Fourteen days later, the cells were continually cultured. Every 3 days, cells and clones were observed microscopically and sub-cultured. After colony formation was completed, the colonies formed by cells were photographed under a microscope and washed three times with PBS. Then, add 1 ml of crystal violet staining solution to each well and stain for 10–20 min. Finally, the six-well plate that formed the clones was scanned.

### Transwell assay

Cal27 and SCC25 cells were added to the upper chamber with 200 μl of serum-free medium. In the lower chamber, 650 μl of medium containing 10% fetal serum was added. In the upper chamber, the rest of the cells were removed with a cotton swab, and those on the surface of the lower chamber were treated with 4% paraformaldehyde for 15 min at room temperature and stained with 0.1% gentian violet for 30 min. Cells from the lower chamber (migrated cells) were imaged under an inverted microscope.

### RNA interference assay

Short hairpin RNA (sh-RNA) sequences of NIFK were synthesized by RiboBio (Guangzhou, China), and the target sequences of sh-NIFK are as follows: sh-NIFK#1: CATCAGTGAAACGGTATAATC, sh-NIFK#2:CGGATGGAGGAGCGATTTAAA. Based on our previous study (30), lentivirus vectors including short hairpin RNA were used for the RNA interference assay.

### Statistical analysis

The Wilcoxon test was used for data that did not conform to a normal distribution. A t-test was used for normally distributed data. Kaplan–Meier survival plots were used to estimate OS between the two groups using the R package “survminer.” Cox regression for survival analysis was performed using the R package “survival.” Time-dependent receiver operating characteristic (ROC) curves were plotted using the R package “timeROC.” All heatmaps were performed *via* the R “pheatmap” package. Data were primarily visualized using ggplot2 R software (v4.1.2). A P-value of <0.05 was considered statistically significant.

## Results

### Identification of NET-enrichment-scores for the patients with HNSCC

**Figure 1:**
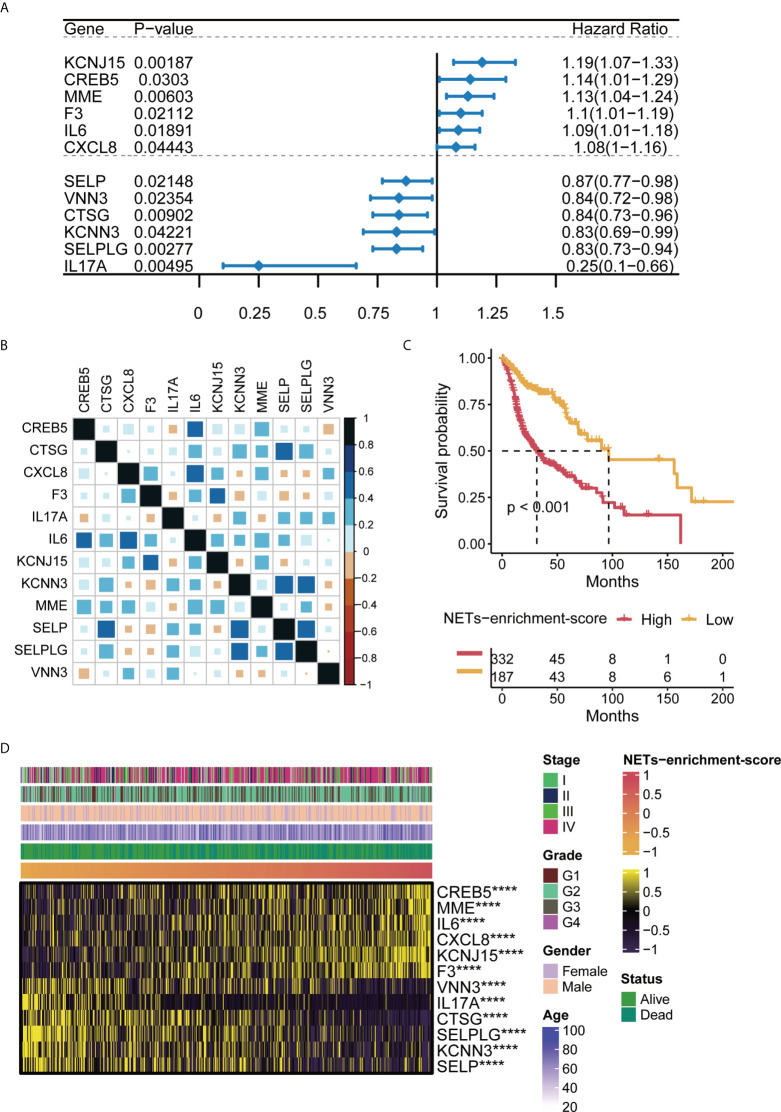
Characteristics of NET-enrichment in HNSC (TCGA). **(A)** Univariate Cox analysis results of 12 prognostic related NET genes in TCGA-HNSCC. **(B)** Correlation map of 12 prognosis-related NET gene sets in TCGA-HNSCC. **(C)** Kaplan–Meier curve showing the correlation of NET-enrichment-score with OS. **(D)** Heatmap showing the correlation of the NET-enrichment-score with 12 prognosis-related NET genes and their clinical features. ****,p<0.001.

Previous studies have applied 69 genes as the neutrophil extracellular trap (NET)-initial biomarkers. To identify a NET-relevant signature for HNSCC, the 69-gene NET-initial biomarkers were applied in the uniCox regression analysis in the TCGA-HNSCC training set, and we found 12 NET-associated genes with prognostic potential in HNSCC, including KCNJ15, CREB5, MME, F3, IL6, CXCL8, SELP, VNN3, CTSG, KCNN3, SELPLG, and IL17A, where the hazard ratio (HR) originated from uniCox regression analysis for each gene was included (**Figure 1A**). The ssGSEA was then applied to the TCGA-HNSCC with the 12 NET-associated genes, and a NETs-enrichment-score was established on the basis of their expression levels. Moreover, the correlation analysis showed that there was a strong correlation between the 12 NET-related genes (**Figure 1B**). Furthermore, the Kaplan–Meier analysis of the 12 NET-related genes showed that the survival of HNSCC patients was inversely correlated with the NET-enrichment-score, implying that HNSCC patients with high levels of the NET-enrichment-score may have a worse prognosis (**Figure 1C**). Finally, a heatmap displayed the correlation between the NET-enrichment-scores and the clinical characteristics of HNSCC samples, referring to the clinical stages, grade, gender, and age (**Figure 1D**). The results showed that each of the NET-associated genes exhibited a strong correlation with NET-enrichment-scores, which correlated with the clinical characteristics of HNSCC patients.

### Establishment of a 7-gene NET related signature for HNSCC

**Figure 2:**
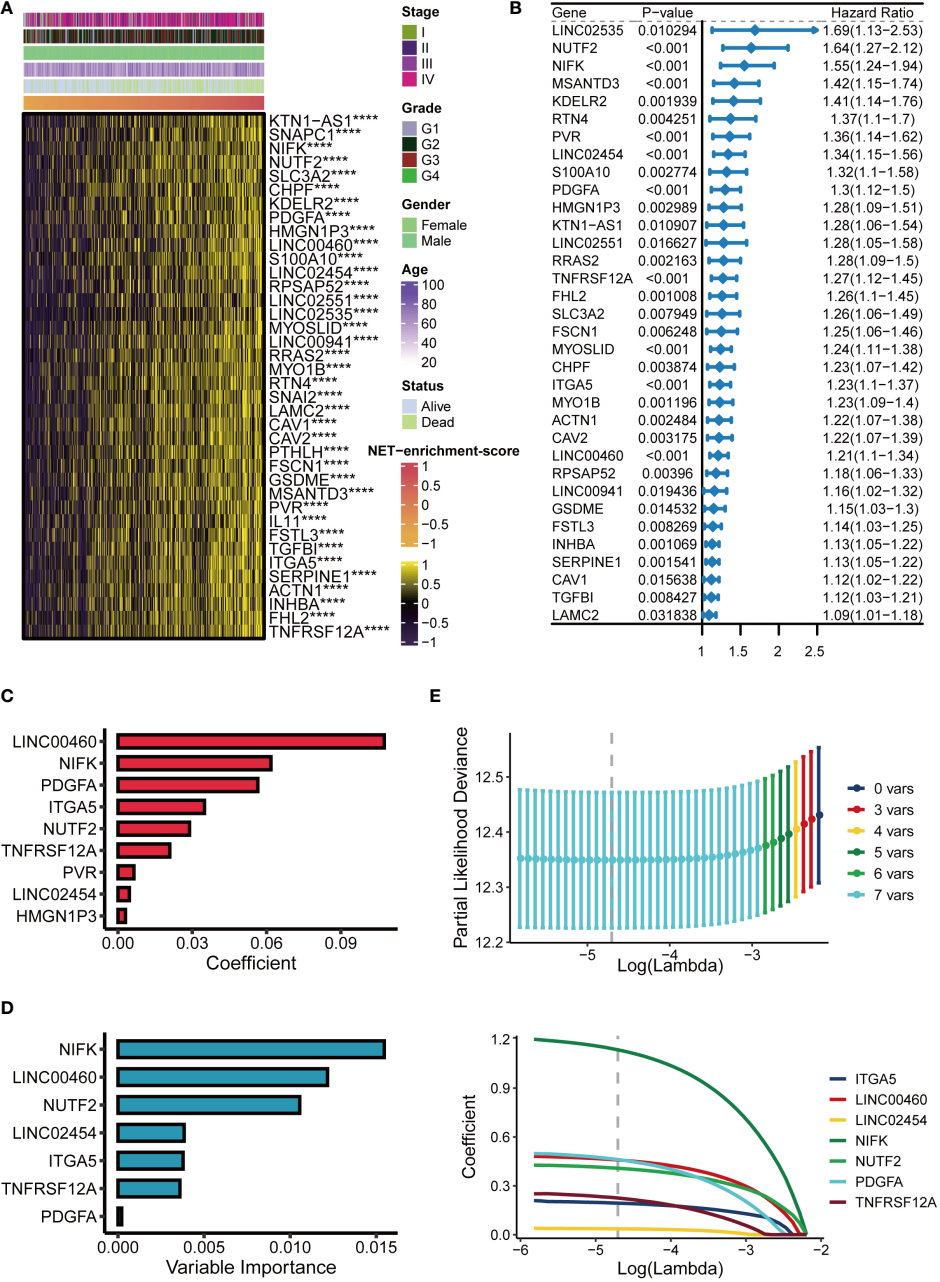
Establishment of the NET score signature. **(A)** Heatmap of 38 NET-related genes significantly positively correlated with NET-enrichment-score. **(B)** Univariate Cox analysis forest plot of 34 prognosis-related NET-related genes. **(C)** The machine learning method CoxBoost further screened NET-related genes (34 dimensionality reduction to 9). **(D)** Machine learning method for survival random forest to further screen NET related genes (reduced from 9 to 7). **(E)** Lasso regression method to calculate NET scores. ****,p<0.001.

Spearman correlation analysis identified 38 NET-related genes were positively correlated with NET-enrichment-scores, based on the criteria with correlation coefficient >0.4 and *P*-value <0.05. Heatmap showed that these 38 NET-associated genes correlated with NET-enrichment-scores and clinical futures of the HNSCC patients (**Figure 2A**). To further screen out the more valuable NET-related genes, univariate Cox regression analysis was performed to further select out 34 NET-related genes of potential prognostic value for HNSCC patients (*P*-value <0.05), and the univariate Cox analysis forest plot showed the HR of each single gene of the 34 NET genes for their prognosis (**Figure 2B**). Moreover, we used machine learning algorithms by applying the R “CoxBoost” to further select out nine NET-related genes (**Figure 2C**). Interestingly, the randomForestSRC (RFC) enabled us to further screen out seven NET-related genes, including NIFK, LINC00460, NUTF2, LINC02454, ITGA5, TNFRSF12α, and PDGFα (**Figure 2D**). Finally, the Lasso regression analysis was performed to calculate new NET-scores using these seven prognostic and NET-related genes based on their estimated regression coefficients (**Figure 2E**). Each estimated regression coefficients of the prognostic-related NET gene were following, 0.1936 ∗ ITGA5 + 0.4588 ∗ LINC00460 + 0.0361 ∗ LINC02454 + 1.1349 ∗ NIFK + 0.4079 ∗ NUTF2 + 0.4611 ∗ PDGF α + 0.2251 ∗ TNFRSF12 α.

### Validation of the NET-scores for clinical predicting the survival in HNSCC

**Figure 3:**
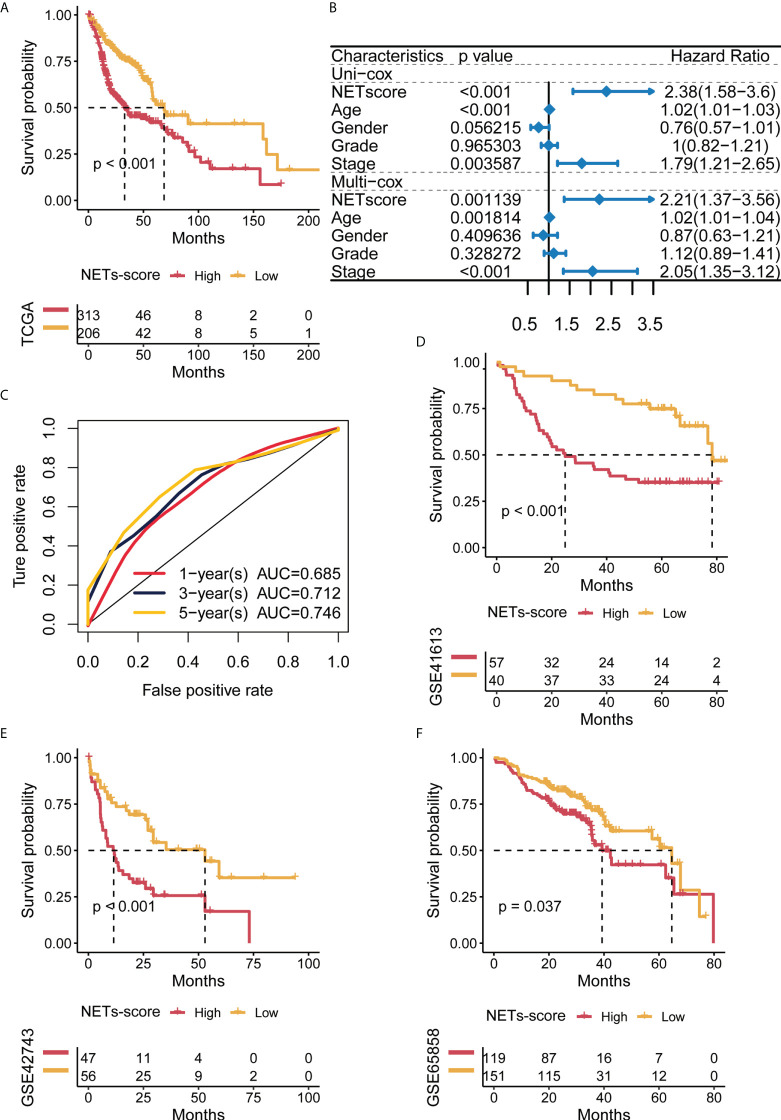
Predictive potential of the NET score for prognosis in HNSCC patients. **(A)** Kaplan–Meier curves of high and low NET scores in TCGA-HNSCC. **(B)** Forest plot of univariate and multivariate Cox regression of NET-score based on TCGA dataset. **(C)** Time-dependent ROC of NET-score in TCGA. **(D–F)** Kaplan–Meier curves of overall survival in HNSCC patients based on an external validation dataset.

To test if the 7-gene NET-score signature predicted the clinical characteristics of the HNSCC patients, Kaplan–Meier analysis revealed that HNSCC patients with higher NET-scores had poor survival curves (**Figure 3A**). Additionally, both univariate Cox and multivariate Cox regression analysis showed that the NET-score of HNSCC patients was an independent risk factor compared with other clinical factors, such as tumor grade and gender (**Figure 3B**). As shown in **Figure 3C**, the time-dependent ROC curves at 1 year, 3 years, and 5 years of OS had AUC values of 0.685, 0.712, and 0.746, respectively, and these results indicated that our NET-score signature was of prognostic value. Furthermore, we used three independent cohorts in the GEO database (ID: GSE41613, GSE42743, and GSE65858) and further verified that HNSCC patients with higher NET-scores had worse prognosis and survival disadvantages (**Figures 3D–F**).

### The NET-score was relevant to distinct genomic alterations of HNSCC patients

**Figure 4:**
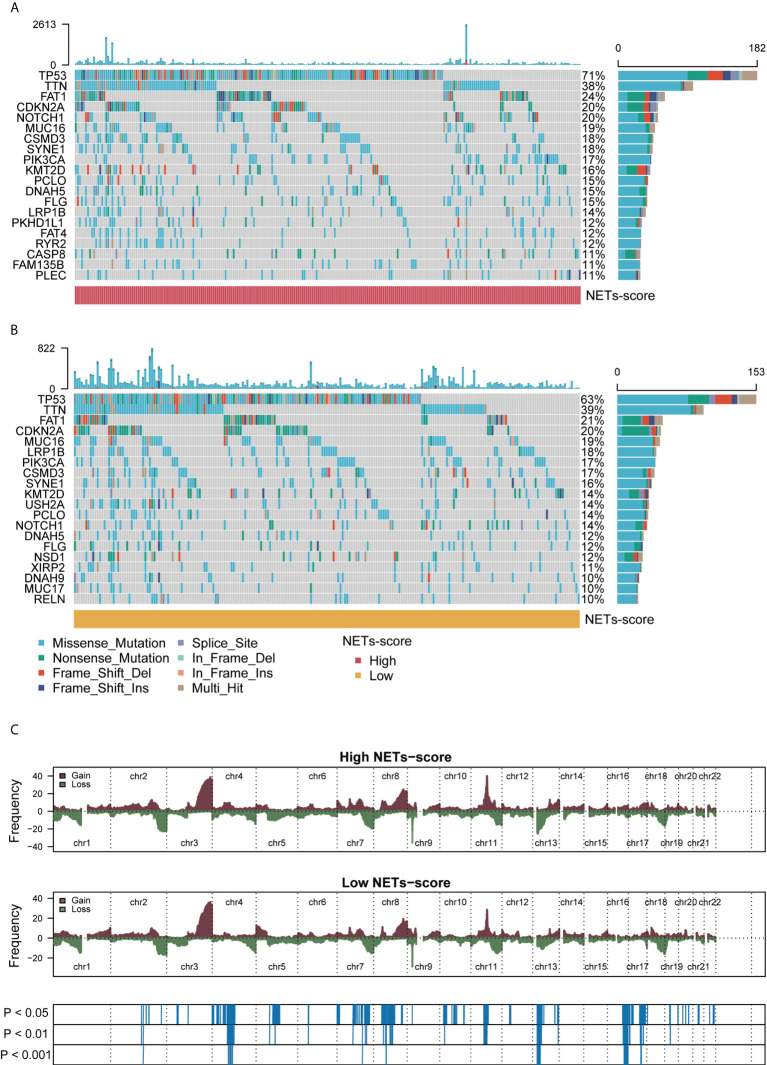
Genomic alterations associated with NET scores in HNSCC samples. **(A,B)** Waterfall plot of somatic mutations in HNSCCs between high and low NET-score groups. **(C)** Copy number changes of HNSCCs between NET-score high and low groups.

To check if somatic mutations are linked to the NET-scores, we conducted the Genomic Identification of Important Targets in Cancer (GISTIC) analysis and the results showed that HNSCC patients with either high or low NET-scores manifested respective somatic mutations, including TP53, PIK3CA, NOTCH1, and MUC17 (**Figures 4A, B**). Nevertheless, HNSCC patients with high NET-scores appeared to have a higher trend of TP53 mutations as compared to patients with low NET-scores, i.e., 71% vs. 63%, respectively (**Figures 4A, B**). Moreover, analysis of the copy number alterations showed that HNSCC patients with either high or low NET scores displayed copy number changes significantly at multiple chromosome loci (**Figure 4C**), probably related to the clinicopathological features of the HNSCC patients.

### The NET-scores are conversely related to immune infiltration for patients with HNSCC in TCGA cohorts

**Figure 5:**
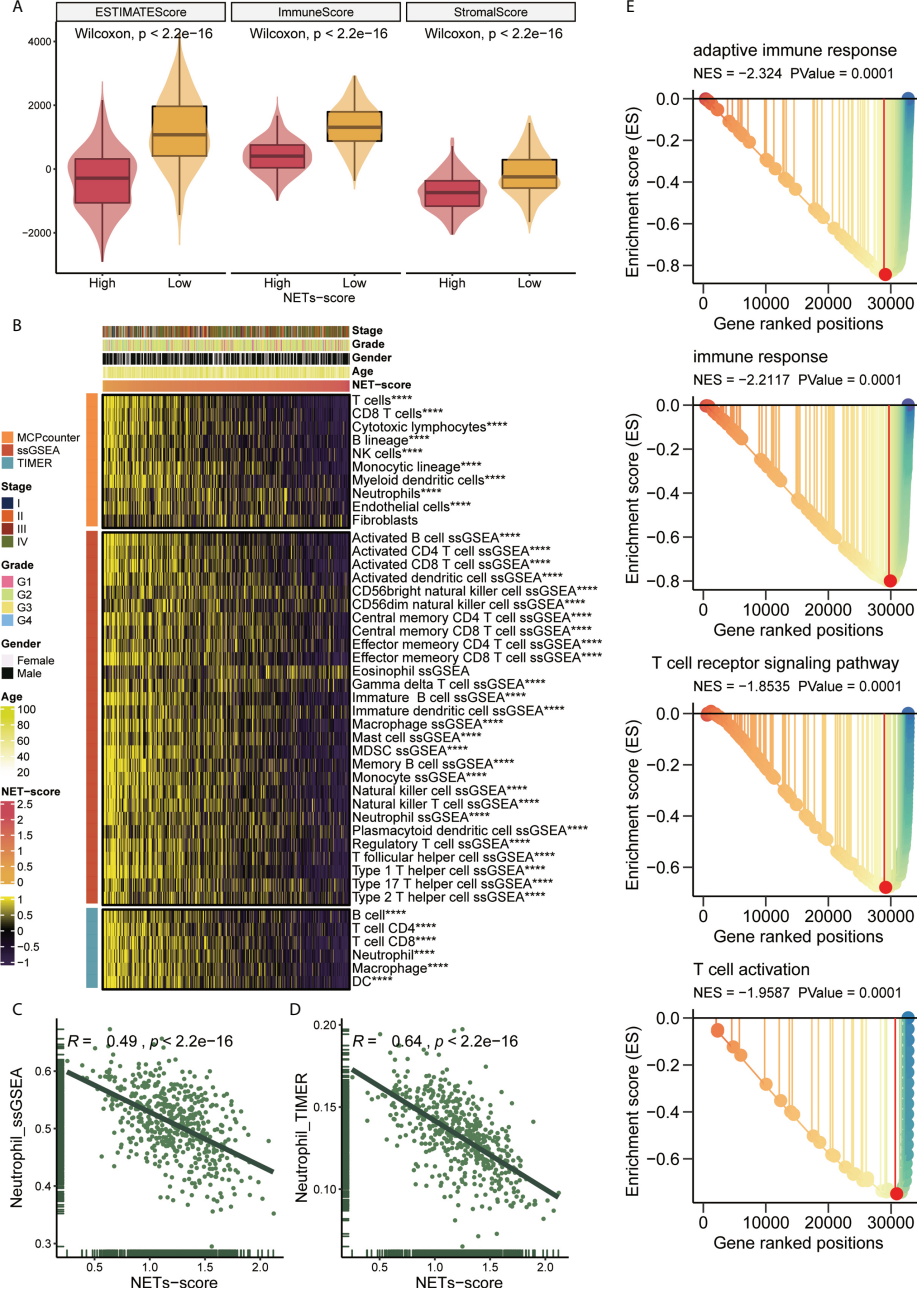
NET scores in relation to immunity in the TCGA cohort. **(A)** Changes in ESTIMATE among HNSCC patients with high and low NET scores. **(B)** Heatmap showing the abundance of infiltrating immune cell populations for different NET scores according to MCPcounter, ssGSEA, and TIMER algorithms. **(C,D)** Correlation of NET scores with Neutrophil_ssGSEA and Neutrophil_TIMER. **(E)** GSEA showing immune related pathways potentially related by NET-score. ****,p<0.001.

To examine the relationship between NET-scores and the immune status in patients with HNSCC in TCGA cohorts, we applied the ESTIMATE algorithm and found that HNSCC patients with low-NET scores had significantly higher ESTIMATE scores, higher immune scores, and higher stromal cells than those with high-NET scores (**Figure 5A**), indicating that the levels of NET scores are reversely correlated with immune status in HNSCC patients. To confirm this phenotype, the MCPcounter, ssGSEA, and TIMER algorithms were independently used to reveal the abundance of immune infiltrating cell populations based on the NET-scores, tumor stages, tumor grade, gender, and age (**Figure 5B**). As a result, the heatmap showed that many immune-infiltrating cells were enriched in HNSCC patients with low NET-scores, including CD8 T cells, cytotoxic lymphocytes, NK cells, and neutrophils (**Figure 5B**). Additionally, the correlation analysis implied that the NET-scores were negatively associated with the abundance of neutrophils in HNSCC patients (**Figures 5C, D**). Moreover, GSEA showed that several important immune-related pathways were more involved in HNSCC patients with low NET-scores, such as adaptive immune response, immune response, T-cell receptor signaling pathways, and T-cell activation (**Figure 5E**). Thus, our data revealed that the NET-scores for HNSCC patients may be highly linked to the tumor immune microenvironment.

### The potential immunotherapeutic and chemotherapeutic response associated with NET-score of HNSCC patients

**Figure 6:**
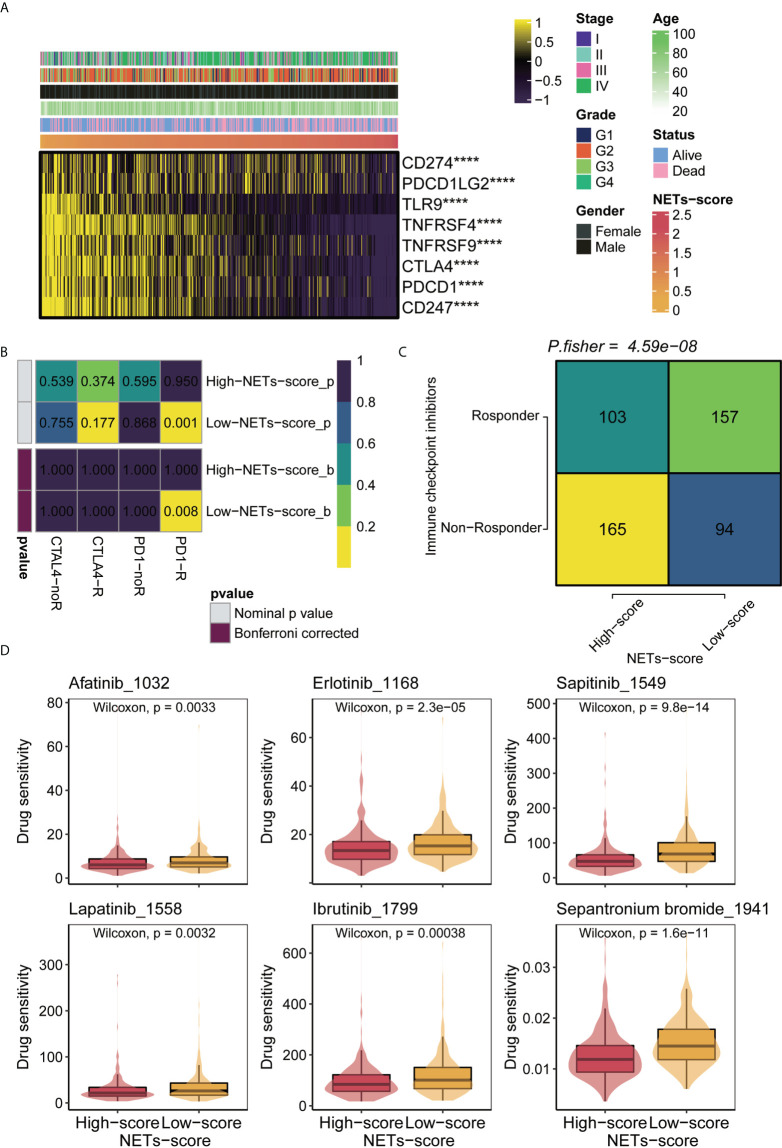
Immunotherapy and chemotherapy of NET scores involved in TCGA-HNSCC. **(A)** Correlation of NET scores and immune checkpoint levels in HNSCC. **(B)** Submap analysis of NET scores in TCGA-HNSCC. **(C)** TIDE analysis of NETs scores in TCGA-HNSCC. **(D)** Boxplots of estimated drug sensitivities for several GDSC chemotherapeutics in the high and low NET scores groups. ****,p<0.001.

Recent developments in immunotherapy, particularly PD-1 inhibitors, have led to the outperformance of traditional chemotherapy in HNSCC at the recurrent and metastatic stages. Using chemo-immunotherapy, chemotherapy interacts with immune cell mechanisms to enhance current cancer treatment strategies (33). To explore the therapeutic responsiveness based on NET-scores, we first checked the correlation between the NET-scores and the immune checkpoint levels in HNSCC patients. The heatmap showed that HNSCC patients with low NET scores tended to have higher levels of immune checkpoints, including CD274 and CTLA4 (**Figure 6A**). Subsequent analysis of drug sensitivity revealed that HNSCC patients with low NET-scores were further enriched in the responders, but not in the non-responders, when immune checkpoint inhibitors were performed (**Figures 6B, C**). In contrast, patients with high NET-scores were probably more non-responders as the immunotherapies were applied (**Figures 6B, C**). Moreover, NET-scores were significantly linked to the targeted therapies, including afatinib, lapatinib, erlotinib, and ibrutinib, indicating that patients with low NET-scores had a better response to the targeted therapies (**Figure 6D**).

### NIFK is a potentially prognostic factor and oncogene for HNSCC patients

**Figure 7:**
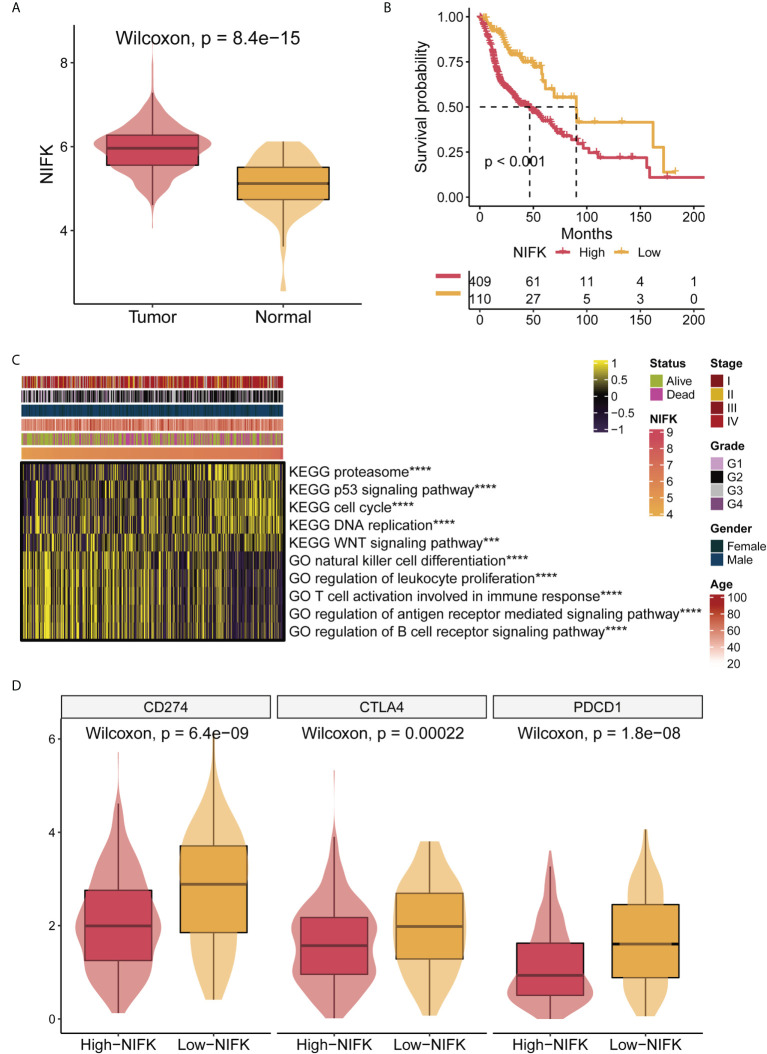
NIFK has an important role in TCGA-HNSCC. **(A)** NIFK levels in HNSCC samples grouped by cancer and para-cancerous status in TCGA. **(B)** Kaplan–Meier curves of TCGA high and low NIFK groups. **(C)** GSVA heatmap showing functional pathways significantly associated with NIFK in TCGA (GO immune-related functions and KEGG cancer-related pathways). **(D)** Association analysis showed that NIFK levels were related to immune checkpoints in TCGA. ***,p<0.001; ****,p<0.0001.

**Figure 8:**
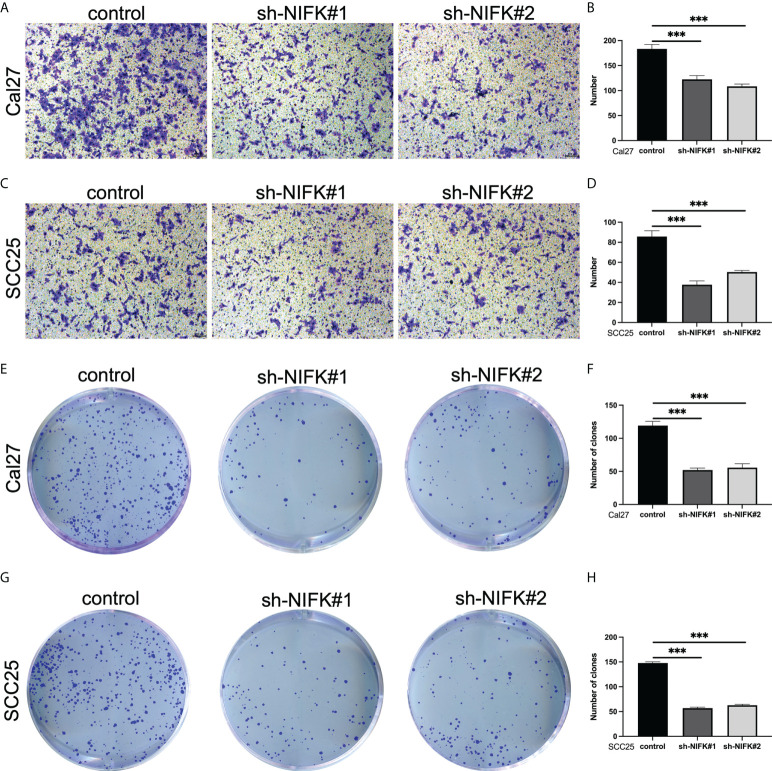
NIFK promotes tumor cell proliferation and metastasis in HNSCC. **(A)** Transwell migration assay in Cal27 cells silenced with control (sh-NC) or NIFK sh-RNA (#1 and #2). **(B)** Quantitative analysis of Transwell assay in Cal27 control and sh-NIFK#1 and sh-NIFK#2 cells. **(C)** Transwell assay in SCC25 control and sh-NIFK#1 and sh-NIFK#2 cells. **(D)** Quantitative analysis of Transwell assay in SCC25 control and sh-NIFK#1 and sh-NIFK#2 cells. **(E)** Plate cloning assay in Cal27 control and sh-NIFK#1 and sh-NIFK#2 cells. **(F)** Quantitative analysis of plate cloning assay in Cal27 control and sh-NIFK#1 and sh-NIFK#2 cells. **(G)** Plate cloning assay in SCC25 control and sh-NIFK#1 and sh-NIFK#2 cells. **(H)** Quantitative analysis of plate cloning assay in SCC25 control and sh-NIFK#1 and sh-NIFK#2 cells. ***P <0.001.

As shown in **Figure 2D**, NIFK was ranked at the top of the 7-gene signature as per their variable importance. NIFK, a nucleolar protein interacting with the fork head associated (FHA) domain of Ki67, may play a role in cell cycle progression and mitosis. However, the function of NIFK in human cancer development is not clear. We therefore examined the expression of NIFK in HNSCC samples and their matched benign or normal tissues, and found that NIFK was significantly expressed in the tumorous samples as compared to their normal counterparts (**Figure 7A**). Further Kaplan–Meier analysis implied that NIFK level is reversely correlated with the survival of HNSCC patients using TCGA cohorts, showing that HNSCC patients with high a level of NIFK had a worse prognosis (**Figure 7B**). In addition, the GSVA heatmap showed several NIFK associated pathways, such as GO immune-related functions and KEGG tumor-related pathways (**Figure 7C**). For example, a high level of NIFK was closely linked to KEGG pathways regulating cell cycle, DNA replication, and proteasome, while a low level of NIFK was associated with immune-related pathways involving natural killer cell differentiation, leukocyte proliferation, and immune response (**Figure 7C**). Subsequent analysis hinted that low levels of NIFK were pertinent to levels of immune checkpoint in TCGA (**Figure 7D**). Additionally, to address the oncogenic role of NIFK in HNSCC, shRNA was used to knock down the mRNA expression levels of NIFK in two HNSCC cell lines that are widely used, such as Cal27 and SCC25. Using Transwell assay in Cal27/SCC25 control, sh-NIFK#1 and sh-NIFK#2 cells, and the results revealed that cell metastasis capacity in Cal27 and SCC25 cells was inhibited by NIFK shRNA (**Figures 8A–D**). Subsequently, to investigate the effect of NIFK on the proliferation of HNSCC, we conducted the plate cloning assay with Cal27/SCC25 control, sh-NIFK#1, and sh-NIFK#2 cells, and the results revealed that colony formation in Cal27 and SCC25 cells was inhibited by NIFK shRNA (**Figures 8E–H**). Thus, these data demonstrate that NIFK is a promising factor for predicting the prognosis of HNSCC patients.

## Discussion

Head and neck squamous cell carcinomas (HNSCCs) are one of the most common malignant cancers (1, 34, 35). The mainstay treatments for HNSCC at the early stage are surgery and/or radiation, which benefit most patients with a good prognosis (4, 36, 37). For HNSCC patients at the advanced or late stages, systematic therapies are recommended, including chemotherapy, targeted therapy, and immunotherapy (12, 38, 39). Although many HNSCC patients at the advanced/late stages initially respond well to these treatments, most of them will eventually fail and progress to recurrent and/or metastatic diseases (2, 4, 40). For example, a randomized phase III clinical trial to compare the efficacy between different strategies of chemotherapy in advanced HNSCC showed that patients treated by cisplatin and fluorouracil (CF) had a median survival of 8.7 months, as compared to patients administered by cisplatin and paclitaxel (CP) with a median survival of 8.1 months (41). Further studies have revealed that cetuximab (a regimen of targeted therapy) plus platinum-fluorouracil chemotherapy significantly improved overall survival (OS) of recurrent or metastatic HNSCC to 10.1 months, as compared to the OS of patients of 7.4 months treated by platinum-fluorouracil chemotherapy alone (42). Recently, immunotherapy has been widely used in a variety of human cancers, including the recurrent or metastatic HNSCC (43, 44). Emerging evidence has demonstrated that the immune checkpoint targeting agent (such as Pembrolizumab) either alone or combined with chemotherapy significantly prolongs OS of the recurrent/metastatic HNSCC with a CPS (the PD-L1 combined positive score) of ≥20, as compared to cetuximab with chemotherapy (45, 46). Therefore, immunotherapy has been recommended as the first-line therapy for recurrent, unresectable, or metastatic head and neck cancers. Nevertheless, either primary or acquired resistance will eventually occur after treatment with immunotherapy. At present, several mechanisms have been proposed to explain these resistant phenotypes, including tumors failing to produce robust T-cell infiltration or tumors excluding T cells. However, the exact mechanisms for the resistance are not completely understood, which will continue to be the future direction in the field (44, 47). Thus, a major hurdle emerging in the field of cancer immunotherapy is the lack of reliable and predictable biomarkers for many cancer patients, including HNSCC (48).

Neutrophil extracellular traps (NETs) participate in the regulation of neutrophil development. They are web-like structures consisting of chromatin and granule proteins (49, 50). NETs have been demonstrated to be linked with different conditions *via* distinct mechanisms, such as inflammation, cell damage, and vascular thrombosis (27, 51). Recently, increasing evidence has shown that neutrophil extracellular traps (NETs) play vital roles in tumor initiation, progression, recurrence, and metastasis (27, 44). In particular, NETs may play essential roles in the tumor microenvironment and are crucial to cancer immunotherapy (52, 53). Additionally, several prognostic models based on NETs have been shown in various human cancers. However, whether NETs are also implicated in HNSCC development and if NETs offer prognostic and predicative value for HNSCC is not understood. Li et al. have shown that oral squamous cell carcinoma (OSCC) patients with late stages (III/IV) had a higher level of NETs compared to early stages (I/II), and NETs dictate a procoagulant phenotype that can be partially dampened by DNase I (54). Moreover, a recent study has identified a 6-gene signature associated with NETs, consisting of LTF, CYBB, SELPLG, GAPDH, ANXA3, and CSF2, which contributes to a clinical prognostic model for HNSCC (55).

To explore the prognostic biomarkers for HNSCC, we have conducted a series of analyses and validations in the current study, and our findings have novel points. First, we systematically applied 69 NET-initial biomarkers using TCGA-HNSCC datasets and identified 12 NET-related genes potentially predictive of prognosis for HNSCC. Second, further analysis has identified seven NET-related genes, including NIFK, LINC00460, NUTF2, and LINC02454, some of which are potentially predictive biomarkers for human cancers. For instance, NUTF2 has been reported to be highly expressed in HNSCC, associated with a poor prognosis and related to immune cells, which may serve as a potential biomarker and target for HNSCC (56–58). Additionally, previous studies have established a 12-gene signature based on the fatty acid metabolism to predict the prognosis of HNSCC, which contain the long non-coding RNA, LINC00460, indicating its predictive role for HNSCC (36, 59, 60). Also, another long non-coding RNA, LINC02454, is linked to predicting the prognosis of thyroid cancer (61, 62). In our study, we set up NET scores based on the seven prognostic-related NET genes, and HNSCC with low-NET scores was related to better prognosis and survival of patients. Importantly, our data hinted that the NET scores for HNSCC patients may be correlated with clinical traits for prognostic prediction (**Figures 3**, **4**). Third, HNSCC patients with low-NET scores had higher immune scores, higher stromal cells, and immune-related pathways, which responded well to immunotherapy and targeted therapies (such as afatinib and lapatinib). Thus, our findings suggest that the seven NET-related gene signatures are predictive of prognosis for HNSCC.

In the current study, we identified that NIFK was highly upregulated in HNSCC patient samples as compared to normal tissues, and HNSCC patients with a high level of NIFK had a worse prognosis and a shortened life span, indicating that NIFK is a potential prognostic biomarker for HNSCC, although further functional validation is required. In **Figure 7**, our characterizations have found that levels of NIFK were related to cell cycle and DNA replication as well as WNT and P53 signaling pathways. In support of the previous reports showing that NIFK is vital for cell cycle progression *via* RNA recognition motif dependent pre-rRNA maturation (63). Nevertheless, how NIFK functions in human cancers is largely unknown. Recent studies have shown that NIFK is indispensable for lung cancer development through Ki-67 dependent cell proliferation and CK1α/β-catenin activated metastasis (64). Whether NIFK plays a similar role in HNSCC development is not clear, and more work is needed for its verification. Our present study has also found that NIFK involvement in HNSCC progression is linked with immune response and immune associated pathways (**Figure 7**), hinting that NIFK is also a potential therapeutic target for immunotherapy for HNSCC, although future work is required to validate this conjecture.

However, our current study has potential limitations. For instance, detailed experimental studies must be added to explore the possible mechanisms of NIFK regulation in HNSCC using cell lines, animal models, and human samples. Furthermore, our seven NET-related gene signatures and our NET scores must be validated in the clinics *via* large-cohort and multicenter studies. Moreover, there exist several gene signatures (including this study) to predict the prognosis of HNSCC. Future studies should be considered to compare the similarities and differences among these signatures and to select the representative targets for HNSCC treatment.

## Data availability statement

The datasets presented in this study can be found in online repositories. The names of the repository/repositories and accession number(s) can be found in the article/**Supplementary Material**.

## Author contributions

QLL conceived and designed the study. QLL and XC drafted the manuscript. QLL and WC did the statistical analysis, supervised by QHL, JM, and XC. All authors have reviewed, critically revised, and approved the manuscript.

## Funding

This study was supported by grants from the National Natural Science Foundation of China (No. 81802973) and the general project of the Natural Science Foundation of Hubei Province (No. 2020CFB844) for QHL. JM was supported in part by the Key Project of the Health Commission of Hubei Province (No. WJ2019Z006). XC was supported, in part, by a grant from the National Natural Science Foundation of China (No. 81602592).

## Acknowledgments

We thank colleagues at the Department of Stomatology and Department of Oncology at Tongji Hospital for support and suggestions for our manuscript. We apologize to the colleagues whose work was not cited due to space limitations.

## Conflict of interest

The authors declare that the research was conducted in the absence of any commercial or financial relationships that could be construed as a potential conflict of interest.

## Publisher’s note

All claims expressed in this article are solely those of the authors and do not necessarily represent those of their affiliated organizations, or those of the publisher, the editors and the reviewers. Any product that may be evaluated in this article, or claim that may be made by its manufacturer, is not guaranteed or endorsed by the publisher.
